# Pediatric Bone Health and Attention-Deficit/Hyperactivity Disorder (ADHD) Stimulants: Raising Awareness of Avascular Necrosis Risk

**DOI:** 10.7759/cureus.86672

**Published:** 2025-06-24

**Authors:** Camila Vicioso, Sri Guttikonda, Prabhjot Singh, Eric Small

**Affiliations:** 1 Department of Orthopedic Surgery, Icahn School of Medicine at Mount Sinai, New York City, USA

**Keywords:** attention-deficit/hyperactivity disorder (adhd), avascular necrosis (avn), avascular necrosis of the hip, developmental and behavioural pediatrics, pediatric sports medicine, stimulant treatment

## Abstract

Avascular necrosis (AVN) is a rare but serious complication of pediatric musculoskeletal injury, traditionally associated with corticosteroid use, trauma, or systemic disease. However, stimulant medications used to treat attention-deficit/hyperactivity disorder (ADHD) may contribute to AVN risk through effects on bone metabolism and vascular tone, including reduced bone mineral density and increased sympathetic activity.

In clinical practice, we observed a case of AVN in a child with ADHD following lower-extremity injury and stimulant use, raising concern about the interplay between the physiological effects of medication and challenges in treatment adherence. Children with ADHD may struggle with non-weight-bearing protocols and follow-up care, which could further elevate the risk of complications.

This article aims to raise awareness of the potential intersection between ADHD stimulant therapy and AVN risk in pediatric patients with orthopedic injuries. We urge clinicians to maintain a high index of suspicion for AVN in this population, particularly when symptoms persist despite appropriate management. Multidisciplinary care and enhanced education for patients and caregivers are critical to ensuring adherence and preventing long-term joint damage.

Further research is warranted to explore the possible association between stimulant use and AVN and to guide best practices for prevention and management.

## Editorial

ADHD stimulants and pediatric bone health: a call for vigilance around avascular necrosis risk

Attention-deficit/hyperactivity disorder (ADHD) is a common neurodevelopmental condition, affecting up to 16% of children [1]. Stimulant medications such as methylphenidate and amphetamines are first-line therapies that can be life-changing, enhancing focus, executive function, and behavioral regulation. However, alongside these well-established benefits, stimulants have been associated with adverse effects on the musculoskeletal system, including reduced bone density, impaired bone healing, and altered bone metabolism [2,3].

Less explored is the potential relationship between stimulant use and avascular necrosis (AVN), a rare but serious condition in which disrupted blood supply leads to bone death, often with permanent joint damage. AVN is most commonly linked to corticosteroid use, trauma, or autoimmune disease, though recent attention includes the role of vasoconstrictive, bone, and metabolic effects of stimulant medications. Stimulants are known to increase sympathetic activity and norepinephrine levels, which can cause vasoconstriction, impair bone vascularity, and increase the risks of vasculopathy [4]. These mechanisms involved with stimulant use are biologically plausible contributors to ischemic bone injury, such as AVN.

These concerns are not merely theoretical. In clinical practice, poor adherence to activity restrictions or missed follow-up appointments in children on stimulant medications has been observed to precede serious orthopedic complications, including AVN in our practice. Children with ADHD may struggle to follow non-weight-bearing protocols or postoperative care plans due to their core challenges [1]. When behavioral vulnerabilities intersect with possible physiological effects of stimulants, the risk of complications such as AVN is amplified.

This perspective aims to raise awareness of the potential intersection between ADHD, stimulant use, and AVN risk, particularly in pediatric patients with lower-extremity injuries. Children on stimulants may require enhanced education, support, and coordinated care to ensure adherence to orthopedic recommendations, especially when AVN is a potential complication following injury. Providers should maintain a high index of suspicion for AVN in these patients, especially when they present with persistent joint pain.

Although a direct causal relationship between stimulant use and AVN has not been established, emerging evidence warrants further investigation. Notably, 80% of studies examining stimulant effects on bone health in children report reduced bone mineral density and content [2], and murine models suggest that methylphenidate directly influences osteoclast activity and bone turnover [3]. Other medications, including corticosteroids, antidepressants, and antipsychotics, have previously been implicated in AVN risk, with one study finding that patients with psychiatric diagnoses (i.e., bipolar disorder) may be at elevated risk compared to neurotypical individuals [5].

Given the widespread use of stimulant medications, it is essential to evaluate their potential impact on pediatric bone health, particularly in the context of musculoskeletal injuries. This article highlights the need for heightened clinical vigilance and targeted research to clarify the relationship between stimulant use and AVN risk and to inform best practices for prevention and management in this vulnerable population.

Practical recommendations for recognizing and managing AVN risk in children with ADHD

Symptoms and Diagnosis

In children, the femoral head is the most common site of AVN. The condition often presents insidiously, with early symptoms including activity-related pain in the hip, groin, or thigh. Over time, this discomfort may become persistent, and progression can lead to an antalgic gait, reduced range of motion, and difficulty bearing weight. While AVN may or may not be preceded by trauma, its hallmark is compromised blood supply to the bone.

In children taking stimulant medications, AVN should be considered earlier in the differential diagnosis due to the known effects of these drugs on bone metabolism and vascular tone. While stimulants may occasionally cause side effects such as muscle stiffness or discomfort, persistent joint pain should prompt a comprehensive evaluation, including medication history, prior injuries, and risk factors such as sickle cell disease, steroid use, or coagulopathies. Because early-stage AVN may not appear on radiographs, MRI is the preferred imaging modality to detect ischemic changes before structural collapse occurs. Prompt referral to a pediatric orthopedic specialist is warranted when AVN is suspected. AVN should remain a key consideration in any child with ADHD who presents with ongoing lower-extremity musculoskeletal symptoms, particularly those engaged in high-impact sports or with known risk factors. Timely diagnosis through clinical vigilance and imaging, followed by early intervention, is essential to prevent long-term complications such as femoral head collapse, joint dysfunction, or chronic pain.

Treatment Considerations

The management of AVN in children focuses on preserving joint integrity, alleviating symptoms, and preventing progression to femoral head collapse. Early-stage AVN is typically managed non-surgically, with interventions such as activity modification, weight-bearing restrictions, and physical therapy to reduce joint stress. Advanced AVN is treated surgically and can have irreversible consequences on bone and joint damage.

However, children with ADHD face distinct challenges in adhering to treatment protocols due to impaired attention, impulsivity, and difficulty following complex instructions, especially when non-weight-bearing restrictions are involved. To improve compliance, clinicians should prioritize clear, consistent communication with both the child and caregivers, reinforcing the importance of early symptom reporting and adherence to physical therapy and activity modifications. Education about the musculoskeletal risks associated with stimulant use, delivered in developmentally appropriate language, can further enhance understanding and cooperation. Involving behavioral health specialists and implementing structured support strategies can also play a critical role in optimizing adherence and preventing long-term joint damage. Finally, an interdisciplinary approach is essential in managing AVN risk among children with ADHD. Pediatricians, orthopedic surgeons, sports medicine providers, psychiatrists, and behavioral health specialists should work collaboratively to ensure comprehensive care, particularly for patients who may struggle with adherence due to attention and executive functioning challenges.

Conclusion

Stimulant medications are widely prescribed for children with ADHD and are known to affect both bone metabolism and vascular tone, two systems central to the pathogenesis of avascular necrosis (AVN). Although a direct causal link between stimulant use and AVN has not been established, the biological plausibility is strong. In clinical practice, we have observed AVN following hip injury where non-adherence to activity restrictions was a contributing factor, highlighting the risks faced by children with ADHD. This overlap underscores the need for heightened clinical vigilance. As stimulant use continues to rise, the potential connection to AVN must not be overlooked. Early recognition, clear communication with families, and multidisciplinary coordination are essential to prevent irreversible joint damage. Importantly, further research is urgently needed to explore this potential association and to inform targeted prevention and treatment strategies for at-risk pediatric populations.
